# E-Spin: A Stochastic Ising Spin Based on Electrically-Controlled MTJ for Constructing Large-Scale Ising Annealing Systems

**DOI:** 10.3390/mi14020258

**Published:** 2023-01-19

**Authors:** Wenhan Chen, Haodi Tang, Yu Wang, Xianwu Hu, Yuming Lin, Tai Min, Yufeng Xie

**Affiliations:** 1 State Key Laboratory of ASIC & System, School of Microelectronics, Fudan University, Shanghai 201203, China; 2 Center for Spintronics and Quantum Systems, State Key Laboratory for Mechanical Behavior of Materials, Department of Materials Science and Engineering, Xi’an Jiaotong University, Xi’an 710049, China

**Keywords:** spintronics, spin-transfer torque magnetic tunnel junctions (STT-MTJ), Ising annealing system, combinatorial optimization problem

## Abstract

With its unique computer paradigm, the Ising annealing machine has become an emerging research direction. The Ising annealing system is highly effective at addressing combinatorial optimization (CO) problems that are difficult for conventional computers to tackle. However, Ising spins, which comprise the Ising system, are difficult to implement in high-performance physical circuits. We propose a novel type of Ising spin based on an electrically-controlled magnetic tunnel junction (MTJ). Electrical operation imparts true randomness, great stability, precise control, compact size, and easy integration to the MTJ-based spin. In addition, simulations demonstrate that the frequency of electrically-controlled stochastic Ising spin (E-spin) is 50 times that of the thermal disturbance MTJ-based spin (p-bit). To develop a large-scale Ising annealing system, up to 64 E-spins are implemented. Our Ising annealing system demonstrates factorization of integers up to $2^{64}$ with a temporal complexity of around $O\left(\sqrt{n}\right)$. The proposed E-spin shows superiority in constructing large-scale Ising annealing systems and solving CO problems.

## 1. Introduction

Combinatorial optimization problems have long been a tangled mess for conventional computers. With the scale of the problem increasing, the consumption of time and hardware resources increases exponentially. Heuristic search techniques, such as the simulated annealing algorithm, have been utilized to search for a solution within a limited time. However, their search effectiveness remains unsatisfactory. It is anticipated that quantum annealing [1,2] would surpass the calculating limit of conventional computers. However, the quantity of Q-bits, the requisite cryogenic environment, and the decoherence generated by the environment’s interface severely restrict the system’s scalability.

Ising annealing was offered as an alternative scenario between classical computing and quantum computing. Ising annealing is a novel non-von Neumann computing architecture that specializes in CO problems [3,4,5,6]. There have been numerous proposals to construct Ising systems with physical entities, including superconductive qubits [7] and degenerate optical parametric oscillators (DOPOs) [3,8,9,10,11]. However, the above methods are hampered by their integration complexity and stringent working conditions. Other methods, such as complementary metal–oxide–semiconductor (CMOS) circuits [12,13,14] and LC oscillators [5], are constrained by circuit area. New devices such as insulator-to-metal phase transition nano oscillators (IMT-NOs) [15] and nano-magnet (p-bit) [16] have been investigated as Ising spins. When manufactured on a wide scale, discrepancies caused by device instability and process variation remain a hurdle. Due to the low computer capability, only 10-bit integer factorization was supported [16]. Therefore, a novel type of Ising spins that are easy to integrate and an efficient Ising annealing algorithm is urgently required.

This paper proposes an electrically-controlled stochastic-MTJ-based Ising spin (E-spin) and an E-spin-based probabilistic Ising annealing algorithm (QFactor). The E-spin has good thermal stability, compact size, true randomness, and high controllability. In addition, the E-spin significantly achieves a 92.2% smaller area and 2% power consumption reduction compared to LFSR [17] based digital Ising spins [16]. Based on the electrically-controlled stochastic MTJ, we introduced a new stochastic Ising annealing algorithm named QFactor. The QFactor outperforms various stochastic or heuristic techniques, including the simulated annealing algorithm and the Ising annealing algorithm, when factoring up to 64-bit integers as a demonstration. The proposed approach is easily extensible to numerous additional CO problems.

## 2. Spin-Transfer Torque Magnetic Tunnel Junctions and E-Spin

### 2.1. Mechanism of the Spin-Transfer Torque Magnetic Tunnel Junctions

**Figure 1:**
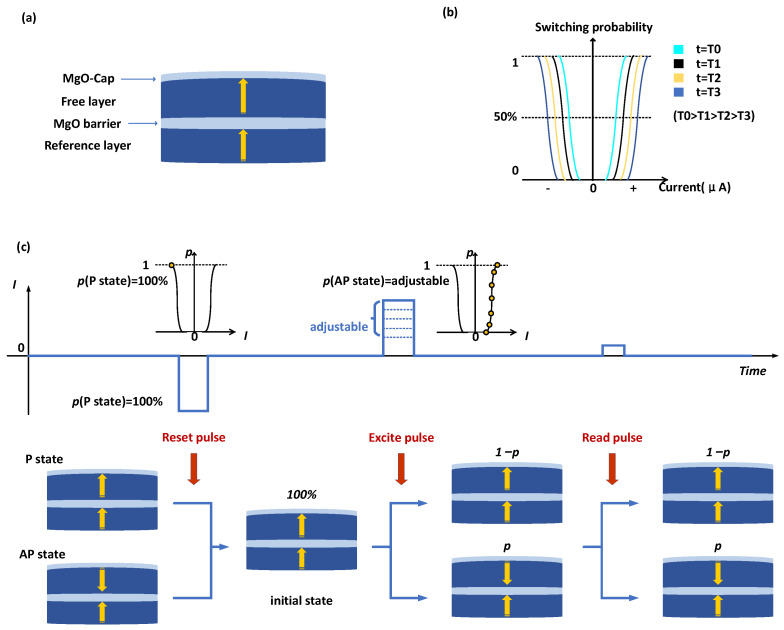
(**a**) Diagrammatic representation of the Double-MTJ employed in this investigation. (**b**) Switching probability of Double-MTJ under various current and pulse duration conditions. (**c**) Process of operation. Pulses for reset, excite, and read are applied in that order.

The spin-transfer torque magnetic tunnel junctions (STT-MTJs) possess the property of stochastic switching [18,19,20], and their switching probability is dependent on the applied current. According to prior research [21,22,23], the switching characteristics of STT-MRAM can be roughly seen as a sigmoidal function of the current magnitude. The schematic diagram of MTJ’s switching probability is depicted in Figure 1b, and it is written as follows:

(1) $$p_{sw}=1-exp\left\{-\frac{t}{\tau_{0}}exp\left[-\Delta {\left(1-\frac{I}{I_{c0}}\right)}^{2}\right]\right\}$$

where $\tau_{0}$, t, Δ, I, and $I_{c0}$ are respectively the attempt time, the duration time of the current pulse, the thermal stability parameter, the applied current, and the critical switching current at 0 K. While t and I are variables that can be changed after fabrication, parameter $\tau_{0}$, ∆, and $I_{c0}$ are process-related parameters and cannot be altered. Applying various current pulses of suitable amplitude and length allows us to change the switching probability [22,24].

The construction of the Double-MTJ employed in this work is depicted in Figure 1a. MgO makes up the tunnel barrier and the cap layer, while CoFeB makes up the free layer. The additional CoFeB/MgO interface raises the energy barrier of STT-MTJ [25].

The process of operation is shown in Figure 1c. First, we must perform the “reset” operation, which involves forcing the MTJ into a low resistance state by applying a strong reverse pulse. We call this state the ‘initial state’. After that, to set the probability of MTJ in a high resistance state as the desired probability p, we must deliver the proper pulse according to (1). This process is called the ‘excite’ operation. A probability sequence with a p probability of a high voltage level is created by carrying out the reset and excite operations in order, then reading the resistance of the MTJ after each excite operation. The pulse given to the MTJ during excite operation determines the probability of a high voltage level in the probability sequence.

### 2.2. Schematic Diagram, Operation Paradigm, and Simulation of the E-Spin

**Figure 2:**
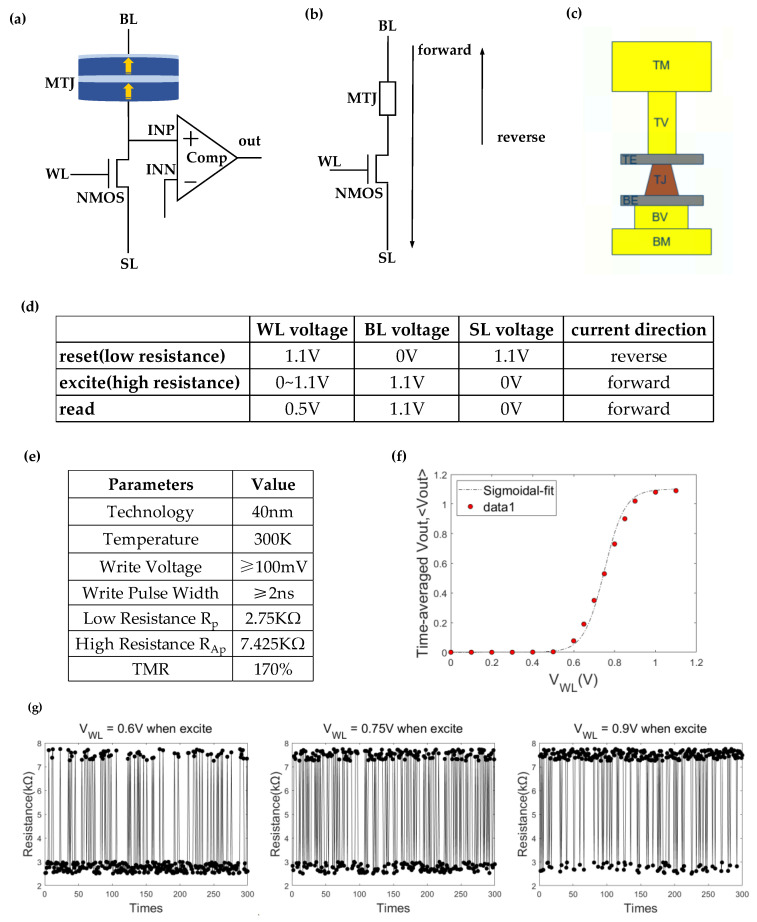
(**a**) Schematic diagram of the E-spin. (**b**) Operation paradigm of the E-spin. (**c**) The layout of the MTJ’s film stack. The technology node of our logic circuit was 40 nm. The MTJ was fabricated on the top layer of the logic circuit. (**d**) The applied voltage for the reset, excite, and read operations. (**e**) The parameters of MTJ were measured at 300 K. The low resistance was 2.75 KΩ and the high resistance was 7.425 KΩ. The TMR (Tunnel Magneto Resistance) was 170%. The switch error rate of the MTJ was correlated with the write voltage and pulse width. The minimum write voltage and pulse width were 100 mV and 2 ns, respectively. (**f**) Time-averaged $V_{out}$, <$V_{out}$>. <$V_{out}$> is the function of the applied input voltage $V_{WL}$ and pulse width. The pulse width is 5 ns in this illustration. The function is fitted to the sigmoidal function. Each data point shown in this figure was averaged for 300 sampling points. (**g**) Time snapshots of the MTJ’s resistance after each excite operation for various input voltage $V_{WL}$ (0.6 V, 0.75 V, and 0.9 V). The MTJ’s resistance was sampled 300 times for each $V_{WL}$.

The proposed E-spin was constructed based on the stochastic STT-MTJ, shown in Figure 2a. 

The n-type metal–oxide–semiconductor (NMOS) transistor is a current source that supplies the electrically-controlled stochastic STT-MTJ with an appropriate amount of current. Depending on the direction and amount of current passing through it, the STT-MTJ may change its state (high or low resistance), altering the voltage of INP. Assuming that the high-resistance MTJ corresponds to $V_{INP}=V_{INP-low}$ and the low-resistance MTJ corresponds to $V_{INP}=V_{INP-high}$, then the voltage of reference INN is set to $(V_{INP-low}+V_{INP-high})/2$. The comparator compares the difference between INN and reference INP and output 1 (0) to distinguish the high (low) resistance state of stochastic MTJ.

The circuit’s current direction is depicted in Figure 2b. The top metal (TM), top via (TV), top electrode (TE), MTJ, bottom electrode (BE), bottom via (BV), and bottom metal (BM) are shown in Figure 2c as the stack structure of the MTJ’s arrangement.

Figure 2d shows the operation paradigm applied to the E-spin. In a single cycle, there are three operational modes: reset, excite, and read. The input of the E-spin ($V_{WL}$) is sufficiently large during the reset period to supply enough current to reset the MTJ to a low resistance state. The direction of the current flowing through the MTJ is reversed during the excite period. As illustrated in Figure 2e,f, a larger $V_{WL}$ corresponds to a higher probability of switching to a high resistance state, in accordance with (1). The comparator, which senses the voltage variation caused by the fluctuation of the MTJ state during the read period, reads out the probability of E-spin.

The output of the ith stochastic MTJ ($m_{i}$) is refreshed during the read period according to:

(2) $$m_{i}=\vartheta \left[\delta \left(I_{i}\right)-r\right]$$

where input $I_{i}$ and output $m_{i}$ can be represented as:

(3) $$m_{i}=V_{out,i}/V_{DD}$$

(4) $$I_{i}=V_{WL,i}/V_{DD}$$

r is a random number with range [0, 1]. δ(x) is the sigmoidal function, and ϑ(x) is the unit step function. As a result, the time-averaged output of the E-spin coincides with the sigmoidal curve, as shown in Figure 2f.

### 2.3. Advantages of the E-Spin

Table 1 shows the characteristics of three MTJ-based random sources, including the spin dice, thermal disturbance MTJ-based spin (p-bit) [16], and our E-spin.

For the spin dice, $P_{sw}$ was set to the fixed value of 50%, while we used the entire domain of the sigmoidal function (see Figure 1) in our design, which broadens the range of its applications.

The temperature, which is determined by the surroundings and operating conditions, has a direct impact on the switching frequency for thermal disturbance MTJ (p-bit) [16]. However, the E-spin, based on Double-MTJ and having a greater thermal stability factor ∆ [25], could counteract the probability skew brought on by temperature variation. Because of this, our proposed electrical operation to adjust probability has better stability. By precisely controlling the current, the dependability and accuracy of the E-spin are considerably enhanced. Additionally, Double-MTJ arrays can be incorporated thanks to the scalability of spin-torque switching, offering significant prospects for novel spintronics-based large-scale integrated circuits [25].

### 2.4. Endurance of the E-Spin

According to the spin operation modes mentioned in Figure 2, repetitive reset, excite, and read operations are required during work. Consequently, the endurance analysis of the spin is required. 

**Figure 3:**
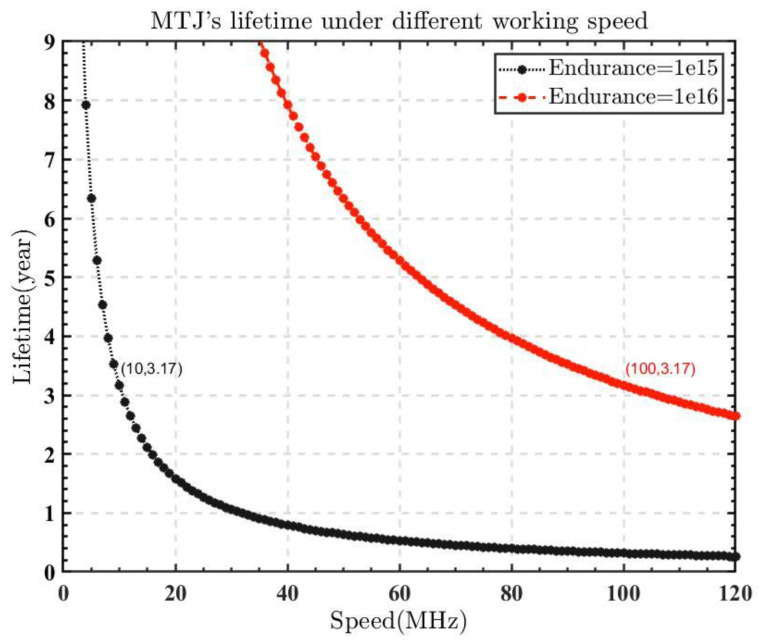
MTJ’s lifetime at various operating frequencies. Under a clock frequency of 10 MHz, MTJ with $1\times 10^{15}$ endurance can operate for 3.17 years.

In the existing non-volatile memories, the endurance of MRAM is an obvious advantage compared to RRAM and PCRAM. The endurance of STT-MTJ with multiple CoFeB/MgO interfaces could theoretically reach up to $10^{15}\sim 10^{16}$, which is higher than traditional MTJ [25]. Constructing spins using such STT-MTJ could reach over three years of lifetime at the working frequency of 10 MHz~100 MHz, satisfying the needs of most usage scenarios. Figure 3 shows the lifetime of such an STT-MTJ at various working frequencies. If the MTJ has an endurance of 1 × 10^15^, it can operate at the frequency of 10 MHz for 3.17 years. The endurance can be increased even more if the SOT-MRAM is included [27]. Because the current is not passed directly through the MTJ in SOT-MRAM, the MTJ is clearly protected, increasing the endurance to almost infinite levels.

## 3. Large-Scale Ising Annealing System for Solving CO Problems Using E-Spins

### 3.1. Steps of Ising Annealing System Solving CO Problems

The Ising system is composed of Ising spins and their interconnections. The Hamiltonian of the Ising system is:

(5) $$H=-\underset{\langle i,j\rangle }{\sum }J_{ij}\sigma_{i}\sigma_{j}-\mu \underset{j}{\sum }h_{j}\sigma_{j}$$

where spin $\sigma_{i,j}\in \left\{+1,-1\right\}$, and $J_{ij}$, $h_{j}$, μ are the coupling parameter between spins i and j, the magnitude of the external magnetic field, and the Bohr magneton, respectively.

By mapping the task function into the Ising Hamiltonian and mapping the best answer to the lowest value of the acquired Ising Hamiltonian, the task can be transformed into searching for the lowest energy state of the Ising system. After the annealing procedure, the system energy will gradually descend and reach the low energy state. At this point, a workable solution is discovered.

### 3.2. Mapping Integer Factorization Problem to Ising Annealing System

An illustration of the combinatorial optimization problem is the integer factorization problem. The integer factorization problem, which is still challenging for traditional computers, is now widely used in the RSA (Rivest–Shamir–Adleman) encryption technique.

According to [28], the Ising Hamiltonian (or system energy, cost function) of the integer factorization problem is:

(6) $$H={\left(X\times Y-F\right)}^{2}$$

where X, Y, and F are odd integers. X and Y can be represented in binary form as:

(7) $$X=\overset{P-1}{\underset{p=0}{\sum }}2^{p}x_{p}$$

(8) $$Y=\overset{Q-1}{\underset{q=0}{\sum }}2^{q}y_{q}$$

with $x_{0}=y_{0}=1$. P and Q donate the number of bits needed to represent X and Y. Suppose *F* is an N-bit integer. Then we have:

(9) N=P+Q

We use E-spins to represent each bit of X and Y. The LSBs (least significant bits) of X and Y are fixed to ‘1’; then, only P−1 and Q−1 E-spins are needed to represent X and Y, respectively. Then we have:

(10) $$X=\overset{P-1}{\underset{n=1}{\sum }}2^{n}m_{n}+1$$

(11) $$Y=\sum_{n=P}^{N-2}2^{n-P+1}m_{n}+1$$

**Figure 4:**
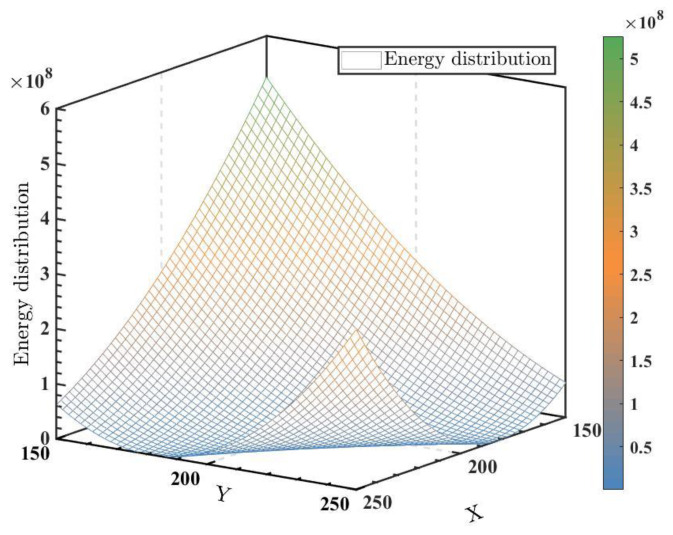
Energy distribution of the system$\;H={\left(X\times Y-45431\right)}^{2}$.

Since the two variables X and *Y* are discrete, H is a discontinuous function. Figure 4 demonstrates an example of the energy distribution of the system $H={\left(X\times Y-F_{i}\right)}^{2}$. Here, $F_{i\;}$= 45,431. It can be inferred from the picture that there are lots of local minimums along the hyperbola $\ell :\;{\left(X\times Y-F\right)}^{2}=0$.

The integer factorization problem differs from other combinational problems in that the former requires the optimal solution, whilst the latter just requires a good enough response. In other words, the output answer must meet higher standards because it will not be legitimate if the product of two factors does not exactly equal the number F.

### 3.3. Stochastic Ising Annealing Algorithm Based on E-Spin

**Figure 5:**
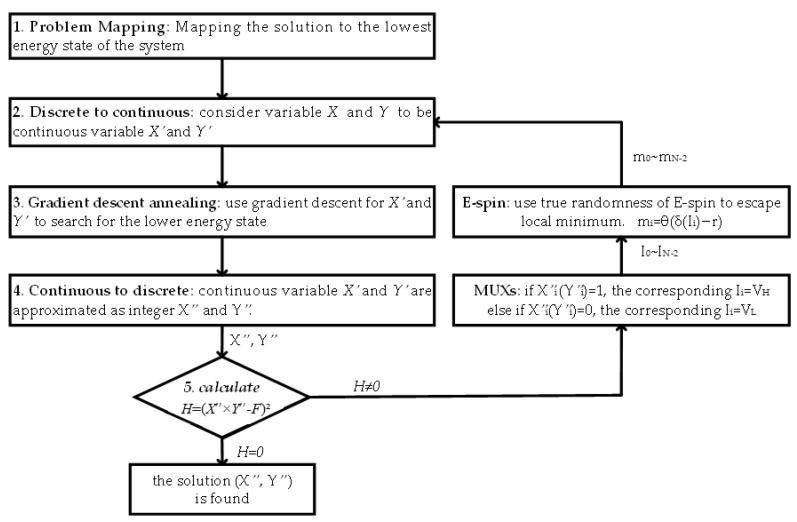
The framework of the proposed Ising annealing algorithm QFactor. *H* = (*X*″ × *Y*″ − *F*)^2^.

Figure 5 demonstrates the flowchart of the proposed QFactor, a stochastic Ising annealing algorithm. The first step is problem mapping. Section 3.2 has shown the procedure of mapping the integer factorization problem to the problem of finding out the minimum value of the discrete function $H={\left(X\times Y-F\right)}^{2}$.

Making the non-continuous function into a continuous function is one of the effective ways to address discrete optimization problems since it speeds up the process of convergence to the local minimum. Therefore, in the second step, the discrete function H was transformed into its continuous function form $H^{\prime }={\left(X^{\prime }\times Y^{\prime }-F\right)}^{2}$, where $X^{\prime }$ and $Y^{\prime }$ are continuous variables. The problem was converted to obtaining the minimum value of the continuous function $H^{\prime }$.

In the third step, gradient descent was employed to reach the local minimum, as deep learning algorithms do in neural networks. Variable $X^{\prime }$ and $Y^{\prime }$ are updated by $X^{\prime }=X^{\prime }+\lambda \cdot \frac{\partial H^{\prime }}{\partial X^{\prime }}$ and $Y^{\prime }=Y^{\prime }+\lambda \cdot \frac{\partial H^{\prime }}{\partial Y^{\prime }}$, where parameter λ is the stride length.

Fourthly, the continuous variable $X^{\prime }$ and $Y^{\prime }$ are approximated as integers $X^{\prime \prime }$ and $Y^{\prime \prime }$.

Lastly, verify that H is equal to 0. H=0 indicates that we have arrived at the right answer.

If H≠0, then we must use the subsequent stages to discover a different solution:

- Map X and Y to the corresponding spin $x_{i}$ and $y_{i}$ using Equations (7) and (8).
- If the calculated $x_{i}$ or $y_{i}$ equals value “1”, the MUX outputs $V_{H}$ to the corresponding $I_{i}$. Then, the E-spin provides a strong likelihood of an output value “1”. The $V_{H}$ is the parameter that can be adjusted. In this experiment, we set $V_{H}$ to be around 0.95 V and $V_{L}$ to be 0.55 V. The random flips of E-spin do not frequently occur, so the gradient descent procedure is dominant in most cases.
- Accordingly, if $x_{i}$ or $y_{i}$ equals ‘0’, the MUX outputs $V_{L}$ to the corresponding E-spin $I_{i}$. Then the E-spin provides a high probability of output value “0”.

Repeat step 2 to step 5 until the correct solution is found.

### 3.4. The Overall Diagram of the Proposed Ising Annealing System

**Figure 6:**
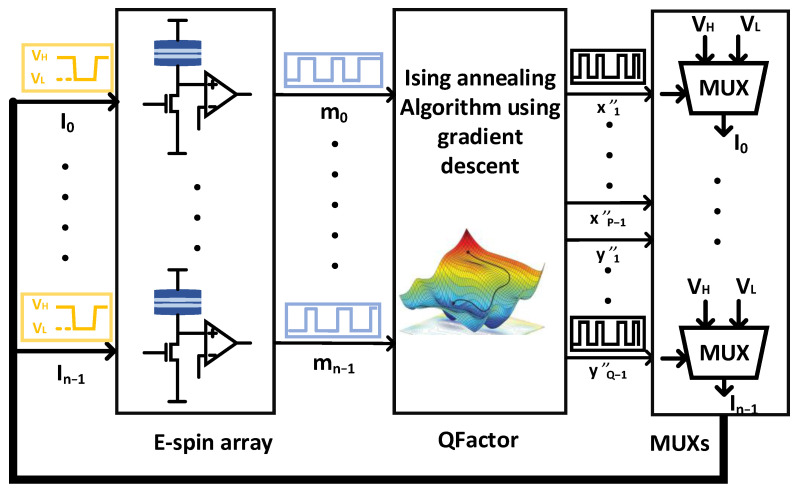
The overall diagram of the proposed Ising annealing system (QFactor). It comprises an E-spin array with 64 E-spins, a digital circuit to implement the QFactor annealing algorithm, and analog MUXs.

The overall diagram of the proposed Ising annealing system is shown in Figure 6. The gradient-descent-based Ising annealing algorithm (QFactor) supplies an input signal for the E-spins through multiplexers (MUXs). If $x_{i}$ (or $y_{i}$) equals ‘1’, then the input of E-spin equals $V_{H}$. Otherwise, if $x_{i}$ (or $y_{i}$) equals ‘0’, then the input of E-spin equals $V_{L}$. Stochastic E-spins generate the sequenced stochastic bit streams according to the MUXs’ output voltage ($V_{H}$ or $V_{L}$).

The key to obtaining a better solution for an Ising annealing system is jumping out of the local optimal solution. Therefore, randomness is required in the annealing procedure to escape the local minimum. A significant difference between QFactor and other Ising annealing algorithms is the production of randomness. The QFactor algorithm fully utilizes the stochastic nature of E-spin, negating the need to use another randomness as the motivation of the annealing procedure.

In addition, QFactor employs randomness differently than traditional heuristic search algorithms, such as simulated annealing, which selects a random number of X and Y for the following annealing cycle. While X and Y are denoted in QFactor as $X=\sum_{n=1}^{P-1}2^{n}m_{n}+1$ and $Y=\sum_{n=P}^{N-2}2^{n-P+1}m_{n}+1$, which means that any random flip of $x_{i}$ will deviate X by the length of $2^{i}x_{i}$.

Except for Ising annealing algorithms and heuristic search algorithms, there are other traditional methods to solve integer factorization problems, such as numerical calculation algorithms. The General Number Field Sieve (GNFS) method is the most effective numerical computation algorithm for solving the integer factorization problem at present. However, because of the operation’s complexity, hardware optimization is challenging. Nevertheless, GNFS is a specific algorithm for a single purpose. The QFactor algorithm provides an easy-to-implement way to solve more general discrete optimization problems.

## 4. Results

### 4.1. Integer Factorization Results

#### 4.1.1. Examples of Factoring Integers

**Figure 7:**
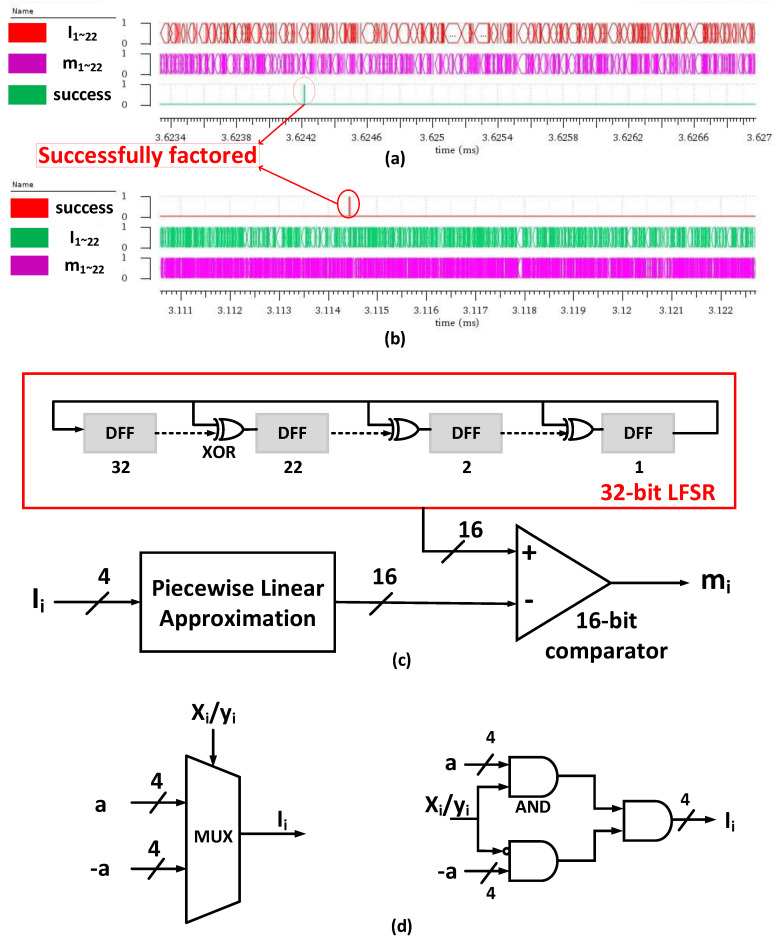
Examples of factoring 24-bit integers 14019841 (**a**) and 14166761 (**b**) using Vivado. $m_{10\sim 1}$ and $m_{22\sim 11}$ represent {$x_{10}$,…, $x_{1}$} and {$y_{10}$,…, $y_{1}$}, respectively. The clock period in this front-end circuit simulation is 500 ns. (**c**) Schematic view of CMOS-based stochastic Ising spin. It comprises a 32-bit LFSR, a piecewise linear approximation module, and a 16-bit comparator. The behavior of the CMOS-based stochastic Ising spin is the same as described in Equation (2). (**d**) Schematic view (left) and circuitry (right) of a digital MUX.

Figure 7a,b show the hardware simulation waveform of factoring 24-bit integers 14019841 and 14166761 using Vivado circuit simulator software. The entire system is simulated using all-digital circuitry for the convenience of larger-scale simulation. The behavior of the E-spin is simulated by the CMOS-based stochastic Ising spin. The 2-way digital MUXs, instead of the analog MUXs in Figure 6, select the input of E-spins.

The schematic view of the CMOS-based stochastic Ising spin is shown in Figure 7c. To better simulate the true-randomness property of the E-spin, we use 32-bit LFSR in our simulation because the 32-bit LFSR counter has a repetition time of up to ($2^{32}$−1) clock periods. We used the high 16 bits of the 32-bit LFSR, which helps to reduce the overall hardware complexity and simulation time. The piecewise linear approximation module approximates the sigmoidal function using 16-segment polylines. The behavior of the MTJ-based E-spin described in Equation (2) is the same as the CMOS-based stochastic Ising spin. Figure 7d shows the schematic view and circuitry of 2-way digital MUXs. During the annealing process, it is proper to reach a balance between gradient descent and random exploration. Therefore, the value of the 4-bit parameter a in Figure 7d is 4 to 6. Suppose that a equals 5, the probability that an E-spin changes its state is $\frac{1}{1+e^{5}}=0.0067$. The probability that at least one E-spin changes its state in the 22 E-spin system is $1-{\left(1-0.0067\right)}^{22}=0.1373$, which indicates that there is a 13.7 percent chance that the E-spins will randomly choose another solution. The value of λ is the other hyperparameter to determine the appropriate stride length for $X^{\prime }$ and $Y^{\prime }$, which is closely related to the bit width n. In this experiment, λ is roughly $6.5\times 10^{-16}$ when n equals 24. a and λ are the only two hyperparameters used in the QFactor, and there is no need to adjust them when factoring different integers with the same n.

Since the LSB of the two factors X and Y is 1, only 22 E-spins are needed. Whenever the system energy $H={\left(X\times Y-F\right)}^{2}$ equals 0, the ‘success’ signal is high. At this time, a successful factorization is reached.

#### 4.1.2. Comparations for Simulated Annealing, Trial Division, and Ising Annealing Algorithm

**Figure 8:**
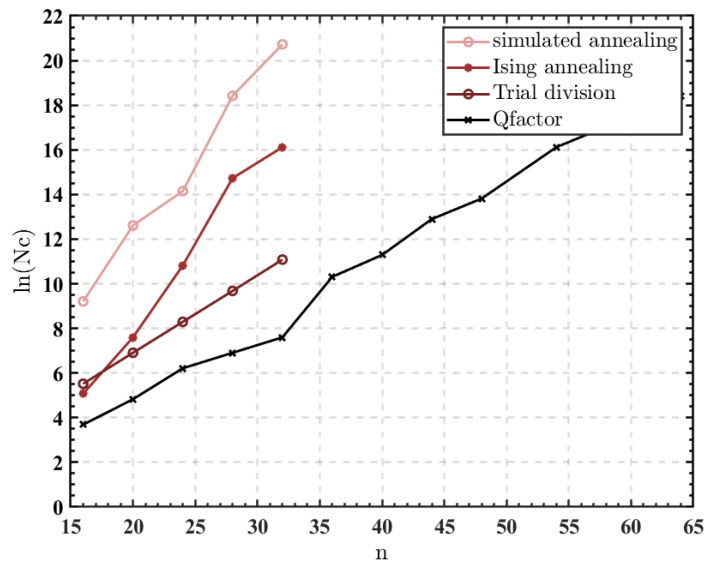
The required cycles to factor n-bit integers. Nc in the Y axis represents the number of required cycles. The four algorithms are simulated using MATLAB software.

Figure 8 shows the averaged cycles needed by the simulated annealing algorithm [12], Ising annealing algorithm [16], trial division [29], and the QFactor to factor integers with n bit widths. The performance of the simulated annealing algorithm was evaluated by mapping the factorization optimization problem into the minimum energy state, carrying out the pseudo-annealing procedure, and selecting the optimal sets of parameters in our trial. Accordingly, the performance of the Ising annealing algorithm was evaluated through our recurrence of the code. The trial division algorithm traverses all numbers from 2 to $\sqrt{n}$ until a factor is discovered. The four algorithms were tested using the exact integers. All values on the graph were tested more than five times.

For instance, the averaged number of cycles needed to factor 32-bit integers using the simulated annealing, Ising annealing, and trial division methods, respectively, is $5\times 10^{5}$, $5\times 10^{3}$, and 33 times greater than the QFactor. Additionally, the QFactor further extended its advantage as the integer bit width increased, remarkably outperforming other stochastic algorithms.

As the integer bit width (n) increases linearly, the size of the search space expands at the rate of $2^{n}$. However, Figure 8 shows that the required cycles of the QFactor only grow at the rate of $2^{\sqrt{n}}$, which not only shortens annealing cycles but also remarkably outperforms other stochastic algorithms. The required cycles of trial division increase at the rate of $2^{\sqrt{n}}$ as well. The QFactor only employs the multiply operation, in contrast to the trial division method’s use of divide operations. The hardware complexity of the divide operation is much higher than the multiply operation, especially as the problem’s size increases. Therefore, implementing the trial division method in hardware is much more complicated than implementing the QFactor.

### 4.2. Analysis Results of the E-Spin

There are several technology roadmaps to implement the stochastic Ising spin. The CMOS-based stochastic Ising spin uses LFSR to build a pseudo-random-number-generator (PRNG). The thermal disturbance MTJ-based spin (p-bit) utilizes thermal-controlled unstable MTJ to realize a true-random-number-generator (TRNG).

**Figure 9:**
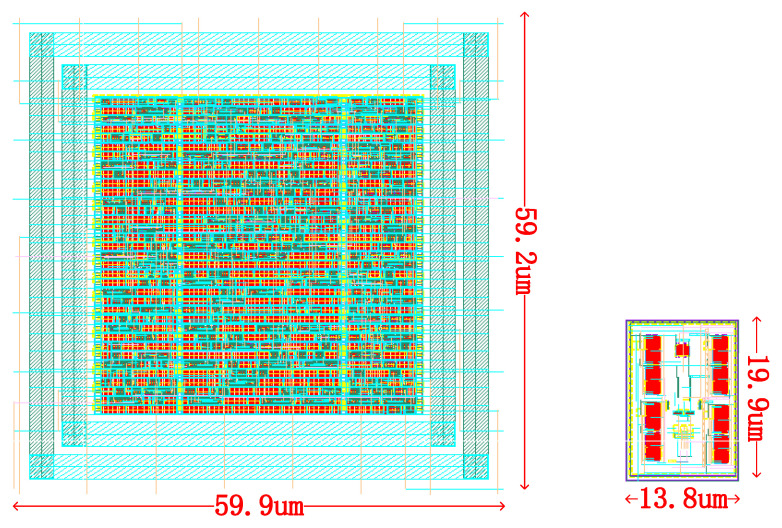
Layout view of a single CMOS-based stochastic Ising spin (left) and a single MTJ-based E-spin (right). The layout of the CMOS-based stochastic Ising spin is automatically generated by EDA (electronic design automation) software DC (Design Compiler) and ICC (IC Compiler). The layout of the MTJ-based E-spin is a customized design using Virtuoso.

Figure 9 shows the layout view of a single CMOS-based stochastic Ising spin and a single MTJ-based E-spin. The area of the CMOS-based stochastic Ising spin is 3546${\;\mu m}^{2}$. The CMOS-based stochastic Ising spin’s power is 142.6 μW at the frequency of 333 MHz, according to the results provided by the Design Compiler.

The MTJ is simulated by a Verilog-A model with 7.425 KΩ high resistance and 2.75 KΩ low resistance. The Verilog-A model is used to simulate the behavior of MTJ under various voltages, which is shown in Table 2.

**Figure 10:**
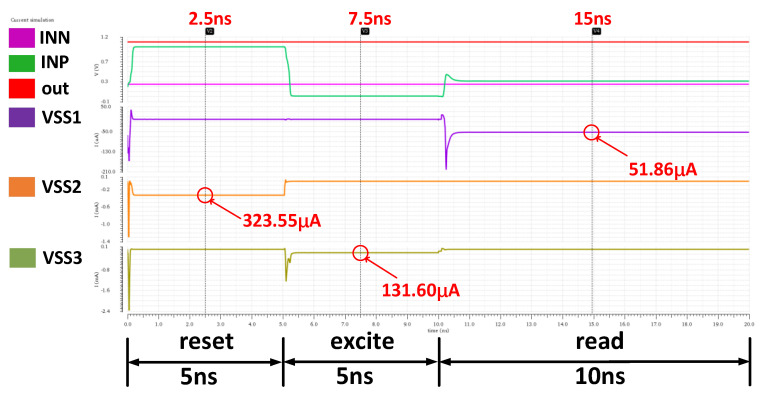
MTJ’s current simulation under reset, excite, and read operation modes.

The current-time simulation of the E-spin is shown in Figure 10. The applied WL, BL, and SL voltages are shown in Figure 2d. In this illustration, $V_{WL,excite}\;$= 0.75 V. Reference voltage $V_{INN}$ is 250 mV. VSS2, VSS3, and VSS1 are the total current under reset, excite, and read operation modes. The reset, excite, and read operations of the stochastic MTJ last 5 ns, 5 ns, and 10 ns, respectively, with currents of 323.55 μA, 131.60 μA, 51.86 μA. Power consumption data (see Table 2) are calculated by multiplying the time-averaged current and VDD (1.1 V).

Table 3 lists the critical indicators of the three stochastic Ising spins.

The primary shortage of LFSR is that it is not a truly random source. The random number generated by an n-bit LFSR will loop for a maximum of $2^{n}$ cycles. That means the size of LFSR must be sufficient to simulate the characteristics of a truly random source. There are some applications that LFSR is incapable of, such as encrypted communication and Monte Carlo simulation.

The primary defect of thermal disturbance MTJ is the intricate voltage offset operation requirement for each bit, which restricts the application in large-scale issues.

The proposed E-spin outperforms the p-bits in stability, speed, and large-scale integration. Firstly, the E-spin adapts the electrical operation, which helps in precise control. Meanwhile, the p-bit uses the thermal-disturbance-controlled MTJ, the frequency of which is influenced by the operating temperature. As a result, the frequency of p-bit is unpredictable, which could cause problems if interconnected with other modules. Additionally, the p-bit is in demand of the voltage offset for each bit. As a result, building an array out of p-bits is challenging.

Secondly, the electrical operation also permits the E-spin to operate with a high frequency. The minimum pulse width of the read/write operation is 2 ns. The frequency of the E-spin could reach higher than 50 MHz. In comparison, the frequency of p-bit is less than 1 MHz. The working frequency of the E-spin is considerably higher.

Lastly, the E-spin could easily integrate with logic circuits. We demonstrate the fabrication of 64 E-spins with logic circuits under the 40 nm technology node, which shows great potential in large-scale integration. Nevertheless, ref [16] only demonstrates eight p-bits, which are selectively chosen from discrete devices. Additionally, the working conditions of each p-bit have to be carefully adjusted. The peripheral circuits for adjusting p-bit working conditions are a considerable expense when manufactured on a large scale.

## 5. Conclusions

This paper proposes a stochastic Ising spin (E-spin) based on the electrically-controlled STT-MTJ. The probabilistic Ising annealing algorithm (QFactor) is based on the E-spins. The E-spin has true randomness, excellent stability, fine control, high frequency, compact size, and is simple to integrate thanks to its electrical operation. Using the gradient descent algorithm and E-spins to escape the local minimum is a novel approach to solving optimization problems. The E-spin achieved 50 times the frequency of p-bit. Up to 64 E-spins are implemented to construct a large-scale Ising annealing system. Factorization of integers up to $2^{64}$ is demonstrated using our Ising annealing system, with a time complexity of roughly $O\left(\sqrt{n}\right)$. This work shows the enormous application potential of the electrically-controlled STT-MTJ, which is also instructive for further research on new computing paradigms.

## Figures and Tables

**Table 1:** Characteristics of the three random sources.

Indicators	Spin Dice [26]	p-bit [16]	E-Spin (This Work)
probability	Fixed	adjustable	adjustable
randomness	TRNG	TRNG	TRNG
operation mode	electric	thermal	electric
reliability	good	poor	good
large-scale integration	easy	hard	easy

**Table 2:** The relationship between the voltage drop of the MTJ and the probability of switching from a low-resistance state to a high-resistance state.

Voltage Drop (mV) ^1^	Switching Probability
[0,144)	0%
[144,171)	7%
[171,212)	20%
[212,275)	32%
[275,342)	48%
[342,428)	66%
[428,584)	81%
[584,718)	93%
[718,731)	98%
[731,1100]	100%

^1^ In this table, the voltage pulse widths are 5 ns.

**Table 3:** Critical indicators of three stochastic Ising spins.

Indicators	CMOS-Based Stochastic Ising Spin	Thermal Disturbance MTJ-Based Spin (p-bit) [16]	E-Spin (This Work)
Area (μm^2^)	1600	NA	280
Speed (MHz)	333	<1	50
Power (μW)	142.6	20	139.7
Technology node	40 nm	NA (discrete)	40 nm
Randomness	PRNG	TRNG	TRNG

## Data Availability

No new data were created or analyzed in this study. Data sharing is not applicable to this article.
